# Effects of Disability Type on the Association between Age and Non-Communicable Disease Risk Factors among Elderly Persons with Disabilities in Shanghai, China

**DOI:** 10.3390/ijerph17155426

**Published:** 2020-07-28

**Authors:** Xichen Wang, Mei Sun, Xiaohong Li, Jun Lu, Gang Chen

**Affiliations:** 1 Department of Health Law and Health Inspection, School of Public Health, Fudan University, Shanghai 200032, China; xichen_326@126.com; 2 China Research Center on Disability Issues at Fudan University, Shanghai 200032, China; sunmei@fudan.edu.cn (M.S.); lixh@fudan.edu.cn (X.L.)

**Keywords:** disability, non-communicable disease risk factor, elderly population

## Abstract

Little is known about differences in the association between age and risk factors of non-communicable diseases (NCDs), according to the disability type in Chinese elderly persons with disabilities. Thus, we examined the effects of these differences in elderly persons with disabilities in Shanghai, China. We evaluated four NCD risk factors (hypertension, hyperglycemia, hyperlipidemia, and overweight) using health data obtained from 20,471 elderly persons with disabilities in 2017. Logistic regression analyses explored differences in the association between age and NCD risk factors according to the disability types, after adjusting for sociodemographic characteristics. We observed significant differences in the association between age and NCD risk factors across disability types; a significant association was observed between older age and higher odds of hypertension (*p* < 0.001) among subjects with a physical disability. However, the prevalence of hypertension did not significantly differ by age in subjects with multiple disabilities. Interventions for elderly patients whose disabilities are more strongly affected by environmental factors should focus more on reduction of subjects’ barriers to activities through improvements in living and environmental adaptability for physical activities.

## 1. Introduction

Persons with disabilities include those who have long-term physical, mental, intellectual, or sensory impairments, which in interaction with various barriers may hinder their full and effective participation in society on an equal basis with others [1]. The World Report on Disability revealed that more than 1 billion persons worldwide—approximately 15.6% of the total global population—lives with some type of disability [2]. Among them, more than 85 million individuals with disabilities live in China [3]. National populations worldwide are aging at unprecedented rates, and the relationship between the trends in global aging and disability is strong and straightforward. Specifically, the risk of disability increases with increasing age [2]. In China, the population of elderly persons (aged ≥ 60 years) with disabilities increases each year by an average of 15.44 million, and by 2050, this population is expected to reach 103 million persons [3]. The higher rates of disability among the elderly reflect the lifetime accumulation of health risks due to disease, injury, and chronic illness.

Not only does disability affect the healthy aging process, but large numbers of elderly persons with disabilities also impose heavy burdens on societies. Disabilities, as well as a lack of egalitarian social policies and accommodations, have multiple and severe impacts on individuals’ lives. Although article 25 of the Convention on the rights of persons with disabilities clearly states that free or low-cost health care services of the same scope, quality, and standard should be provided to persons with disabilities [1]. Individuals with disabilities face high rates of poverty and health challenges and are among the most marginalized groups in society [2]. Risk factors for non-communicable diseases (NCDs), such as uncontrolled hypertension, impaired fasting glucose control, hyperlipidemia, and overweight, may cause diabetes or cardiovascular disease, which can co-exist with a disability and increase the health burdens on affected populations [4]. The aging process may also increase the risks of some chronic diseases, and this risk may be magnified in persons with disabilities. Accordingly, aging would be expected to have a more serious impact on the physical health of elderly persons with disabilities [5]. Attention and research on NCDs risk factors of elderly persons with disabilities can provide evidence and basis for providing them with reasonable and appropriate health services.

Recent studies have revealed an increase in the risk of NCD with age [6,7]. Regardless of disability, increased aging further restricts an elderly person’s ability to participate in activities and increases their sensitivity to environmental NCD risk factors [8]. The physical characteristics of elderly persons with disabilities differ considerably from those of their healthy counterparts. Specifically, the former group has a worse health foundation, less mobility, and an increased vulnerability to chronic diseases [9]. Accordingly, researchers conducting monographic studies of health in persons with disabilities must pay attention to the effects of the type of disability on health-related risk factors [10,11].

Despite these identified differences, China does not sufficiently distinguish elderly persons with disabilities from their relatively healthy peers. In most regions of China, elderly individuals are classified as a single group without stratification by age differences or an understanding of differences in disability types or self-care abilities. This lack of distinction is particularly apparent in the fields of chronic disease management and long-term care. In the Chinese health sector, effective prevention of NCD risk factors requires suitable measures tailored for elderly persons with disabilities. An understanding of the differences in the association between age and NCD risk factors according to disability type in an elderly population would attract attention to the characteristics of subjects with disabilities. This focus would facilitate the implementation of more appropriate interventions.

In this study, we used the health examination data of 20,471 elderly persons with disabilities in Shanghai, China, to examine differences in the association between age and NCD risk factors among elderly persons with disabilities according to the disability type. Our study had the following aims: (1) to compare the sociodemographic characteristics and prevalence of NCD risk factors among elderly subjects stratified by age; and (2) to compare and explain differences in the effects of disability type on NCD risk factors in elderly subjects with disabilities in different age groups.

## 2. Materials and Methods

### 2.1. Data Source and Ethics Statement

Shanghai has provided free yearly health examination services for persons with disabilities since 2004. These examinations are conducted voluntarily. Health examination services were accessed by 34,829 persons or 6.79% of all persons with disabilities in Shanghai between 1 January and 31 December, 2017. We defined “elderly” according to the definition set forth by the World Health Organization as an individual of age ≥ 60 years [12]. For the analysis, we selected four important NCD risk factors, hypertension, hyperglycemia, hyperlipidemia, and overweight, which have considerable effects on public health, especially in elderly populations.

After eliminating the data of persons aged < 60 years old (*n* = 14,043) and those with missing data for the selected variables (*n* = 315), 20,471 elderly persons with disabilities were included in our analyses. This sample accounted for almost 6.32% of the total population of elderly persons with disabilities in Shanghai. The health examination records and sociodemographic information of these individuals were collected by the Shanghai Disabled Persons’ Rehabilitation Comprehensive Information Platform (SHDPRCIP), which was established by the Shanghai Disabled Persons’ Federation. The institutional review board (IRB) of the Fudan University School of Public Health (IRB #2015-08-0563) authorized this study protocol. All participants provided informed consent when they participated in the health examination.

### 2.2. Dependent Variables

Hypertension was defined as a systolic blood pressure ≥140 mm Hg and/or diastolic pressure > 90 mmHg [13]. Hyperglycemia was defined as a fasting blood glucose level ≥ 6.1 mmol/L, based on the Chinese Prevention and Treatment Guideline for Type 2 Diabetes (2013) [14]. Hyperlipidemia was defined as a total cholesterol level ≥5.2 mmol/L or triglyceride level ≥ 1.7 mmol/L, according to the Chinese Adults’ Prevention and Treatment Guidelines for Dyslipidemia (2016) [15]. Overweight was defined according to the recommended guideline of a body mass index (BMI) ≥24 kg/m^2^ for the Chinese population [16]. All dependent variables were categorized as binary outcomes.

### 2.3. Independent Variable

Elderly subjects with disabilities were divided into five equidistant age groups corresponding to 60–64, 65–69, 70–74, 75–79, and ≥80 years.

### 2.4. Covariates

Demographic characteristics, including sex (men or women), residence permit (rural or urban), education level (elementary school or below, middle school, high school, or college or higher), and marital status (never married, married, divorced, or widowed), were regarded as covariates in this study. The disability type and disability severity were also included as covariates in this study. According to the Classification and Grading Criteria of Disability (GB/T 26341-2010) [17], the category of disability types included hearing disability, speech disability, visual disability, physical disability, intellectual disability, mental disability, and multiple disabilities. Subjects with hearing disability or speech disability were grouped [16]. Intellectual disability referred to the level of intelligence, which is significantly lower than that of ordinary people, and accompanied by the obstacles of adaptive behavior. Mental disability referred to the existence of cognitive, emotional, or behavioral barriers that affect their daily life and social participation. The difference between mental disability and intellectual disability lies in the fact that mental disability may have the same intelligence as ordinary people, and its obstacle is more reflected in suffering from a certain mental disease. Multiple disabilities referred to subjects with two or more types of disabilities. Disability severity was classified into four levels, using the related function scores for every disability type, according to standard Chinese criteria [17]. Levels 1 and 4 corresponded to most and least serious disability levels, respectively.

### 2.5. Statistical Analysis

The SPSS Statistics 22.0 software package (IBM Corporation, Armonk, New York) was used for all data analyses. The age distribution by frequency was calculated for all demographic factors, disability types, and disability severity groups. Differences in these variables were analyzed using Pearson’s chi-square test. Next, we fitted logistic regression models to assess and explore the associations between age and NCD risk factors across disability types. After adjusting for covariates in these models, including sex, residence permit, education level, marital status, and disability severity, we refitted logistic regression models stratified by disability types to assess differences in the abovementioned associations across disability types. Participants aged 60–64 years were set as the reference group, and their data were used to estimate odds ratios (ORs) and 95% confidence intervals (CIs) for the other four age groups. We used a forest plot to present the results of our stratified analysis. A *p* value < 0.05 was considered to indicate statistical significance.

## 3. Results

Table 1 presents the participants’ sociodemographic and disability characteristics according to age group. The average age (±SD) in the overall sample was 66.59 ± 5.37 years, and 52.15% of the sample was men. Participants aged 60–64 years accounted for 42.81% of the sample, the largest proportion, while only 2.89% were aged ≥ 80 years. Most participants had an urban residence permit (84.83%). Overall, 49.14% of the sample had a middle school education. Furthermore, 10.07% of participants aged 75–79 years had a college or higher degree, compared to 9.63% of those aged ≥ 80 years. Most participants, 89.26%, were married, whereas only 7.22% were divorced or widowed and 3.51% of participants had never married. Physical disability was the most frequent disability type, affecting half of the participants (54.72%), followed by visual disabilities (25.31%). Regarding disability severity, most participants were classified as level 4 or 3, accounting for 51.63% and 21.93% of the study sample, respectively.

**Figure 1:**
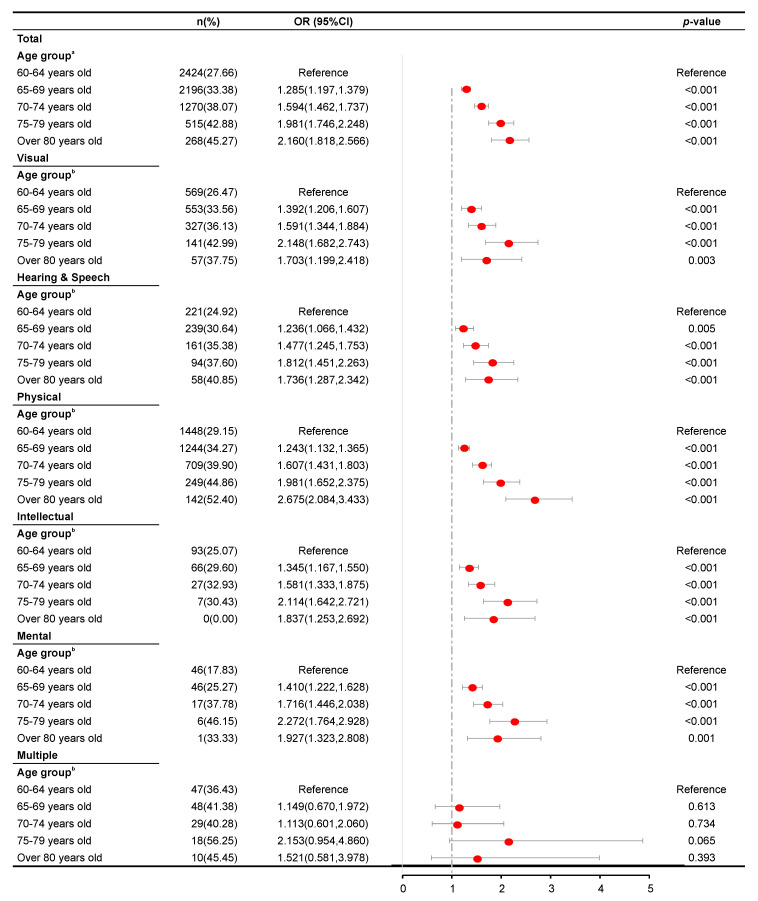
Results of a logistic regression analysis of hypertension according to age group across disability types. ^a^ Adjusted for sex, residence permit, education level, marital status, disability type, and disability severity. ^b^ Adjusted for sex, residence permit, education level, marital status, and disability severity.

**Figure 2:**
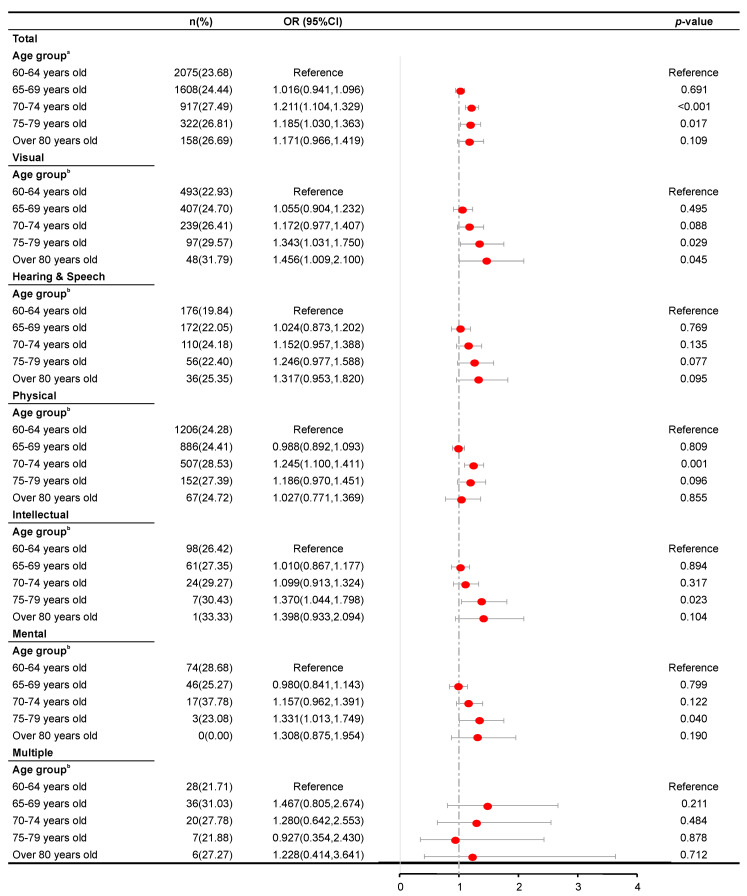
Results of a logistic regression analysis of hyperglycemia according to age group across disability types. ^a^ Adjusted for sex, residence permit, education level, marital status, disability type, and disability severity. ^b^ Adjusted for sex, residence permit, education level, marital status, and disability severity.

**Figure 3:**
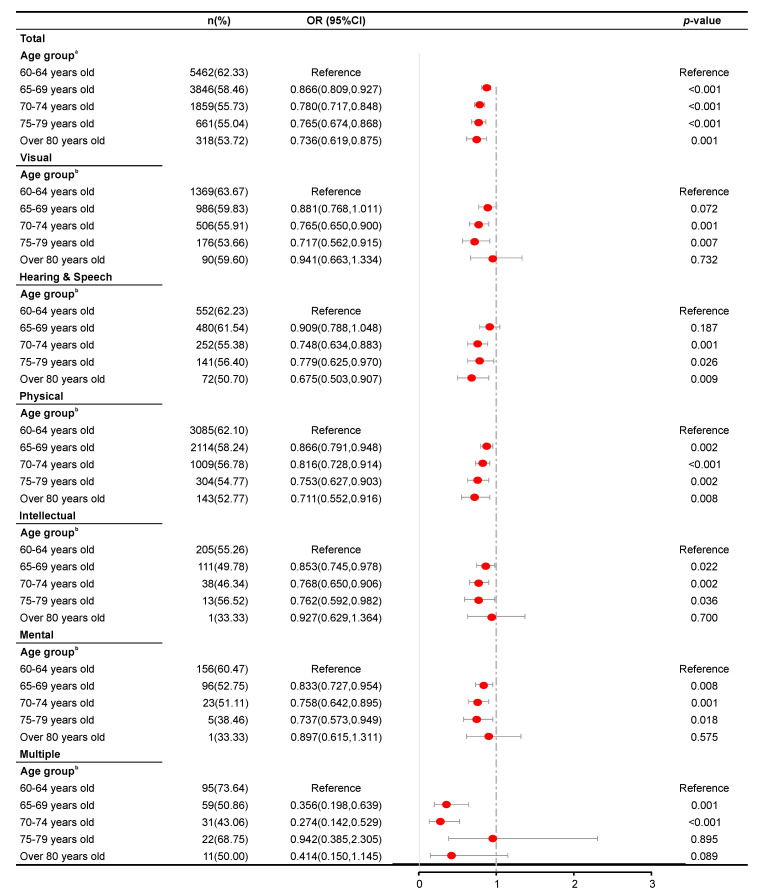
Results of a logistic regression analysis of hyperlipidemia according to age groups across disability types. ^a^ Adjusted for sex, residence permit, education level, marital status, disability type, and disability severity. ^b^ Adjusted for sex, residence permit, education level, marital status, and disability severity.

**Figure 4:**
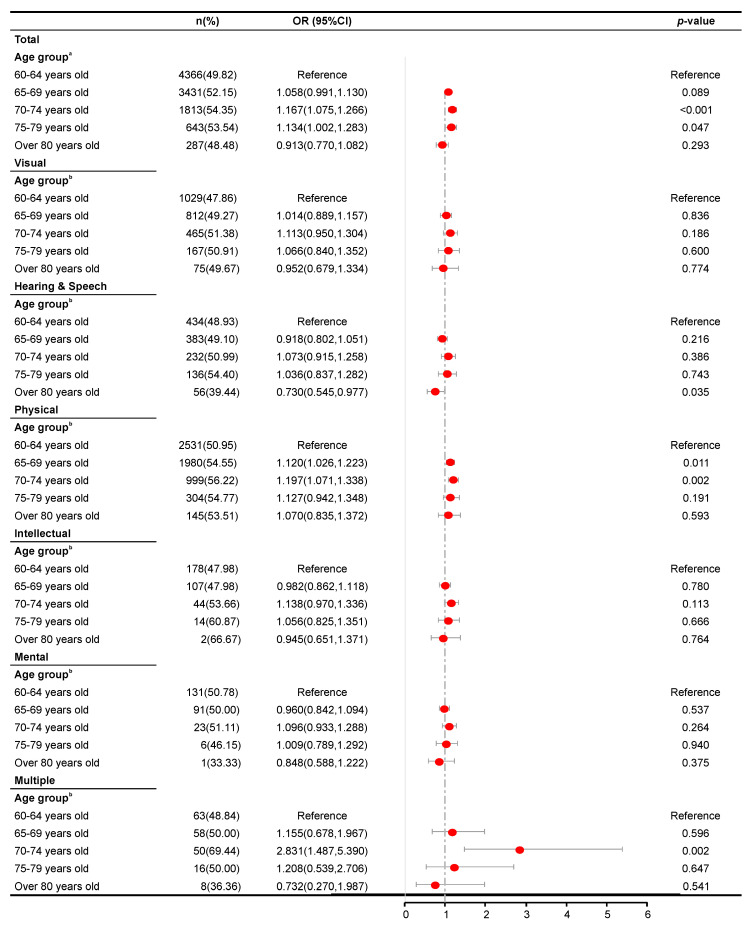
Results of a logistic regression analysis of overweight according to age group across disability types. ^a^ Adjusted for sex, residence permit, education level, marital status, disability type, and disability severity. ^b^ Adjusted for sex, residence permit, education level, marital status, and disability severity.

Figure 1, Figure 2, Figure 3 and Figure 4 present the results of the regression analysis. Overall, when compared with an age of 60–64 years, ages of 65–69 (OR = 1.285, *p* < 0.001), 70–74 (OR = 1.594, *p* < 0.001), 75–79 (OR = 1.981, *p* < 0.001), and ≥80 years (OR = 2.160, *p* < 0.001) were significantly associated with a higher odds of developing hypertension. However, the same groups were significantly associated with lower odds of developing hyperlipidemia, with respective ORs of 0.866, 0.780, 0.765, and 0.736 (*p* < 0.001 for ages of 65–69, 70–74, and 75–79 years and p = 0.001 for ≥80 years). Moreover, the ages of 70–74 and 75–79 years were significantly associated with higher odds of developing hyperglycemia (OR = 1.211, *p* < 0.001 and OR = 1.185, *p* = 0.017, respectively) and overweight (OR = 1.167, *p* < 0.001 and OR = 1.134, *p* = 0.047, respectively).

An analysis stratified by disability type revealed analogous patterns of association between age and the risk of hypertension among patients with either visual, hearing and speech, physical, intellectual, or mental disabilities. However, such patterns were not observed among those with multiple disabilities (*p* = 0.613, 0.734, 0.065, and 0.393 in the 65–69, 70–74, 75–79, and >80 years groups, respectively). Moreover, an association between age and hyperglycemia was not observed in subjects with hearing and speech disability and those with multiple disabilities (*p* = 0.769, 0.135, 0.077, and 0.095 and *p* = 0.211, 0.484, 0.878, and 0.712 in the 65–69, 70–74, 75–79, and ≥80 years groups, respectively). For all disability type stratifications, a significant association was found between age and risk of hyperlipidemia, as revealed by analogous patterns in the analysis, where visual disability had, *p* = 0.001 and *p* = 0.007 in the 70–74 and 75–79 years groups, respectively. Hearing and speech disability, *p* = 0.001, 0.026, and 0.009 in the 70–74, 75–79, and ≥80 years groups, respectively; physical disability, *p* = 0.002 in the 65–69 and 75–79 years groups, and *p* < 0.001 and *p* = 0.008 in the 70–74 and ≥80 years groups, respectively. Intellectual disability, *p* = 0.022, 0.002, and 0.036 in the 65–69, 70–74 and 75–79 years groups, respectively; mental disability, *p* = 0.008, 0.001, and 0.018 in the 65–69, 70–74, and 75–79 years groups, respectively; multiple disabilities, *p* = 0.001, and *p* < 0.001 in the 65–69 and 70–74 years groups, respectively). Only hearing and speech disability, physical disability, and multiple disabilities were found to be associated significantly with the risk of overweight (hearing and speech disability, *p* = 0.035 in the ≥80 years group; physical disability, *p* = 0.011 and *p* = 0.002 in the 65–69 and 70–74 years groups; multiple disabilities, *p* = 0.002 in the 70–74 years group.

## 4. Discussion

Our study not only explored the associations between age and four NCD risk factors, but also investigated potential differences in these associations with respect to different disability types. In Shanghai, older elderly subjects with disabilities tended to be more educated, but were more likely to be divorced or widowed, compared to relatively younger (60–64 years) subjects. Although the prevalence rates of hypertension, hyperglycemia, and overweight were higher among the oldest-old adults in our sample, relative to the younger subjects, a similar pattern was not observed for hyperlipidemia. Our study design differs from that of previous studies, which used age only as a simple control variable in analyses of NCD risk factors among elderly persons with disabilities. However, our study findings suggest that the effect of age may vary by disability type.

We observed that older subjects had relatively higher risks of hypertension, hyperglycemia, and overweight, compared to younger subjects. This finding was consistent with the results of previous studies [18,19,20]. Nevertheless, we found that an older age significantly reduced the risk of hyperlipidemia, possibly because Chinese elderly persons with disabilities are more subjectively dependent a low intake of dietary fats. For example, elderly persons with disabilities may control their intake of high-fat foods such as ribs or marbled meat, due to income restrictions and experiences with self-health protection.

In our study, we identified some differences in the associations between age and NCD risk factors after classifying the elderly according to disability types. For example, no significant associations between age and the risks of hypertension and hyperglycemia were observed among elderly subjects with multiple disabilities, who generally tend to have a poor capacity for self-care ability and a poor health status [21]. Moreover, compared to subjects with single disabilities, elderly persons with multiple disabilities are more severely affected by their physiological deficiencies and environmental factors and are severely restricted with respect to exercise and other behaviors that can reduce the risks of NCDs, regardless of age. These effects of multiple disabilities are persistent and prevalent among the elderly in all age groups. Therefore, the association between age and the risk of NCD risk factors was reduced in the elderly with multiple disabilities, compared to that in the elderly with other disability types.

The elderly with a single disability type may gain more rehabilitation benefits from the increasing use of barrier-free facilities and assistive devices, compared with the elderly with multiple disabilities [22,23,24]. The elderly with a single disability (e.g., the elderly with only visual disabilities) could have approximately the same benefits, which can compensate their dysfunction from the external environment. For example, an elderly adult with only a hearing disability may recover with just the use of a hearing aid. Accordingly, the association between age and the risk of NCDs might increase. However, the elderly with multiple disabilities cannot gain the same benefits as gained by the elderly with a single disability from the external environment and, thus, have a more difficult pathway to recovery. The potentially more severe effects of a worse environment or history of serious illness relative to age might also explain the association between age and the prevalence of NCD risk factors in elderly subjects with multiple disabilities.

In our study, we did not observe significant associations between age and the risk of hyperglycemia among subjects with hearing and speech disabilities. Possibly, hyperglycemia causes or aggravates these disabilities, and this reverse relationship might completely offset the association between age and the risk of hyperglycemia. Moreover, as diabetes most frequently affects middle-aged and elderly persons, hearing impairment may be attributed to age-related deafness [25]. However, related studies reported that the rate of hearing disability among patients with diabetes could be as high as 70% [26]. A professional hearing examination may reveal an existing sensorineural hearing impairment, even in approximately 40% of diabetic patients who do not self-perceive hearing impairment [27]. Therefore, elderly persons may not realize that persistent hyperglycemia and hearing impairment exist in a vicious circle, even at younger ages. Consequently, age may not mediate the association between hearing and speech disability and hyperglycemia in elderly persons.

In contrast, significant associations between age and the risk of hyperlipidemia were observed in all disability type groups, including multiple disabilities, suggesting that older age has an inhibitory effect on the risk of hyperglycemia regardless of disability type. The avoidance of high fat, high-cholesterol foods, and the regular practice of exercise has long been considered effective means of preventing hyperlipidemia [28,29]. In elderly subjects with disabilities, reduced dietary intake, and more regular dietary habits may more effectively reduce the risk of hyperlipidemia in older age groups. The effect of diet on the blood lipid profile is even more significant than that of exercise in elderly persons with multiple disabilities, most likely because these persons find it difficult to exercise.

We did not observe significant associations between age and the risk of overweight in elderly subjects with visual disability, intellectual disability, and mental disability. Reduced exercise activity and motivation for physical activity may increase the risk of overweight, especially in the oldest-old subjects [30]. However, elderly persons with visual disability face more environmental barriers to activity, compared with those with physical disability [31]. The former group is subjectively less inclined to participate in exercise, even when barrier-free facilities have been well established. The existing data strongly suggest that environmental and subjective factors may increase the risk of overweight more than age, among Chinese elderly adults with visual disability. Moreover, elderly adults with intellectual or mental disability face greater fears of becoming lost or experiencing an accident and are more confined to their families due to a reduced ability for self-care. Additionally, many of these people had lost their athletic abilities at a young age [32]. Regardless of age, these people often require assistance from wheelchairs and family members when away from home. This objective situation may reduce the strength of the association between age and the risk of overweight in this subpopulation of elderly persons. Moreover, the risk of overweight was significantly lower among persons aged ≥ 80 years than among 60-year-olds with hearing and speech disability but was significantly higher among those aged 70–74 years with multiple disabilities, compared to their 60-year-old peers. We suspect that the observed association between age and the risk of overweight was magnified by other environmental factors.

In general, our results suggest that differences in disability types should be considered an essential distinguishing factor between elderly persons with disabilities. Efforts to prevent hypertension, hyperglycemia, and overweight should continue to focus on relatively older persons with disabilities. However, efforts to prevent hyperlipidemia should focus on younger elderly or middle-aged persons with disabilities who are just entering old age. Some interventions can target both the general elderly population and their peers with disability types known to have a low impact on the association between age and NCD risk factors, thus enhancing social support while reducing discrimination and the costs associated with differentiated approaches. However, interventions for elderly persons with disabilities that are more strongly affected by environmental factors than by age should focus more on improvements in living and environmental conditions for physical activities. Moreover, approaches to chronic disease management must consider sociological and family factors [33]. Rehabilitation training should be provided for elderly persons with disabilities, regardless of age, because improvements in the psychological and physiological functions of these people would improve their quality of life and behaviors; thus control the prevalence of NCD risk factors.

### Limitations

This study had some limitations. First, the design was cross-sectional, and therefore, causality could not be inferred. Second, we did not control for many societal and environmental factors, such as the causes and duration of disability, health insurance coverage, and behavioral factors, such as the use of related medications and level of physical activity. Future studies must consider more covariates, as this will provide a more complete overview of barriers to social participation that the elderly person face due to disability type differences. Finally, this study collected health examination data from the SHDPRCIP. Some elderly persons with disabilities may have been unwilling to participate in an initial health examination, leading to potential selection bias. Nevertheless, these concerns were somewhat mitigated by the large sample size and the use of objective indicators of disabilities.

## 5. Conclusions

This study showed differences in the associations between age and various NCD risk factors across six disability types. Our findings provide evidence for the need for targeted public policies and strategies for NCD risk factor prevention and management in elderly persons with different disability types and age groups who reside in Shanghai. Policymakers should consider the type of disability and degree of aging more thoroughly when designing programs to target NCD prevention and intervention among the elderly. Precise interventions for disability characteristics and improved barrier-free environments should be developed to promote general health among elderly persons with disabilities better.

## Figures and Tables

**Table 1:** Sociodemographic and disability characteristics of the sample stratified by age group.

Characteristics	Total		60–64 Years		65–69 Years		70–74 Years		75–79 Years		≥80 Years		*p*
	*n*	%	*n*	%	*n*	%	*n*	%	*n*	%	*n*	%	
**Sex**													
Male	10675	52.15	4453	50.82	3453	52.49	1796	53.84	648	53.96	325	54.90	0.008
Female	9796	47.85	4310	49.18	3126	47.51	1540	46.16	553	46.04	267	45.10	
**Residence permit**													
Rural	3105	15.17	1412	16.11	959	14.58	467	14.00	184	15.32	83	14.02	0.017
Urban	17366	84.83	7351	83.89	5620	85.42	2869	86.00	1017	84.68	509	85.98	
**Education level**													
Elementary school or lower	4835	23.62	1417	16.17	1657	25.19	1015	30.43	472	39.30	274	46.28	<0.001
Middle school	10059	49.14	4399	50.20	3783	57.50	1349	40.44	362	30.14	166	28.04	
High school	4577	22.36	2684	30.63	857	13.03	695	20.83	246	20.48	95	16.05	
College or higher	1000	4.88	263	3.00	282	4.29	277	8.30	121	10.07	57	9.63	
**Marital status**													
Never married	719	3.51	392	4.47	206	3.13	92	2.76	21	1.75	8	1.35	<0.001
Married	18273	89.26	7783	88.82	5938	90.26	2999	89.90	1059	88.18	494	83.45	
Divorced or widowed	1479	7.22	588	6.71	435	6.61	245	7.34	121	10.07	90	15.20	
**Disability type**													
Visual	5182	25.31	2150	24.53	1648	25.05	905	27.13	328	27.31	151	25.51	<0.001
Hearing and Speech	2514	12.28	887	10.12	780	11.86	455	13.64	250	20.82	142	23.99	
Physical	11201	54.72	4968	56.69	3630	55.18	1777	53.27	555	46.21	271	45.78	
Intellectual	702	3.43	371	4.23	223	3.39	82	2.46	23	1.92	3	0.51	
Mental	501	2.45	258	2.94	182	2.77	45	1.35	13	1.08	3	0.51	
Multiple	371	1.81	129	1.47	116	1.76	72	2.16	32	2.66	22	3.72	
**Disability severity**													
Level 1	1758	8.59	656	7.49	554	8.42	316	9.47	157	13.07	75	12.67	<0.001
Level 2	2632	12.86	1139	13.00	842	12.80	418	12.53	135	11.24	98	16.55	
Level 3	5512	26.93	2381	27.17	1836	27.91	834	25.00	317	26.39	144	24.32	
Level 4	10569	51.63	4587	52.35	3347	50.87	1768	53.00	592	49.29	275	46.45	
